# Supplementary material for: JunB regulates homeostasis and suppressive functions of effector regulatory T cells
Source: Nat Commun. 2018 Dec 17;9:5344. doi: 10.1038/s41467-018-07735-4 (PMC6297218; doi:10.1038/s41467-018-07735-4)
Supplement: Supplementary file 3 — Reporting Summary [file 41467_2018_7735_MOESM3_ESM.pdf]

nature research

Corresponding author(s): Hiroki Ishikawa

Reporting Summary

Nature Research wishes to improve the reproducibility of the work that we publish. This form provides structure for consistency and transparency in reporting. For further information on Nature Research policies, see Authors & Referees and the Editorial Policy Checklist.

Statistical parameters

When statistical analyses are reported, confirm that the following items are present in the relevant location (e.g. figure legend, table legend, main text, or Methods section).

n/a	Confirmed	
☐	☒	The exact sample size (n) for each experimental group/condition, given as a discrete number and unit of measurement
☐	☒	An indication of whether measurements were taken from distinct samples or whether the same sample was measured repeatedly
☐	☒	The statistical test(s) used AND whether they are one- or two-sided<br>*Only common tests should be described solely by name; describe more complex techniques in the Methods section.*
☒	☐	A description of all covariates tested
☐	☒	A description of any assumptions or corrections, such as tests of normality and adjustment for multiple comparisons
☐	☒	A full description of the statistics including central tendency (e.g. means) or other basic estimates (e.g. regression coefficient) AND variation (e.g. standard deviation) or associated estimates of uncertainty (e.g. confidence intervals)
☐	☒	For null hypothesis testing, the test statistic (e.g. F, t, r) with confidence intervals, effect sizes, degrees of freedom and P value noted<br>*Give P values as exact values whenever suitable.*
☒	☐	For Bayesian analysis, information on the choice of priors and Markov chain Monte Carlo settings
☒	☐	For hierarchical and complex designs, identification of the appropriate level for tests and full reporting of outcomes
☒	☐	Estimates of effect sizes (e.g. Cohen's d, Pearson's r), indicating how they were calculated
☐	☒	Clearly defined error bars<br>*State explicitly what error bars represent (e.g. SD, SE, CI)*

Our web collection on statistics for biologists may be useful.

Software and code

Policy information about availability of computer code

Data collection	N/A
Data analysis	Flow Jo_V10 was used for analysis of flow cytometric data. Excel 2016 and GraphPad Prism 7.01 was used for data analysis. Bowtie2 was used for ChIP-seq and RNA-seq analysis. Homer 4.10 was used for ChIP-seq analysis. Tophat2 .2.1 and cuflinks 2.2.1. were used for RNA-seq analysis.

For manuscripts utilizing custom algorithms or software that are central to the research but not yet described in published literature, software must be made available to editors/reviewers upon request. We strongly encourage code deposition in a community repository (e.g. GitHub). See the Nature Research guidelines for submitting code & software for further information.

Data

Policy information about availability of data

All manuscripts must include a data availability statement. This statement should provide the following information, where applicable:

- Accession codes, unique identifiers, or web links for publicly available datasets
- A list of figures that have associated raw data
- A description of any restrictions on data availability

All other data supporting the findings of this study are available from the authors on request.

Field-specific reporting

Please select the best fit for your research. If you are not sure, read the appropriate sections before making your selection.

☒ Life sciences ☐ Behavioural & social sciences ☐ Ecological, evolutionary & environmental sciences

For a reference copy of the document with all sections, see nature.com/authors/policies/ReportingSummary-flat.pdf

Life sciences study design

All studies must disclose on these points even when the disclosure is negative.

Sample size	Sample sizes were determined according to the basis of previous publications in the immunology field and our previous experience. In most of the experiments, 3 - 8 samples per condition was considered as enough to identify the deferences. Exact sample sizes were indicated in the figure legend.
Data exclusions	No exclusion criteria was used.
Replication	Data are representative of two or more independent experiments with similar results (statistical significance).
Randomization	No randomization method was used.
Blinding	Histology scoring and weight measurement were performed in blind conditions.

Reporting for specific materials, systems and methods

Materials & experimental systems

n/a	Involved in the study	
☐	☒	Unique biological materials
☐	☒	Antibodies
☒	☐	Eukaryotic cell lines
☒	☐	Palaeontology
☐	☒	Animals and other organisms
☒	☐	Human research participants

Methods

n/a	Involved in the study	
☐	☒	ChIP-seq
☐	☒	Flow cytometry
☒	☐	MRI-based neuroimaging

Unique biological materials

Policy information about availability of materials

Obtaining unique materials	All the materials used are available without restrictions.

Antibodies

Antibodies used	For flow cytometry analysis and fluorescence-activated cell sorting (FACS), the following antibodies were used: anti-CD3 (17A2, Biolegend, 1:400), anti-CD4 (GK1.5, Biolegend, 1:100 or 1:400), anti-CD8 (53–6.7, Biolegend, 1:400), anti-CD25 (PC61, Biolegend, 1:400), anti-CD44 (IM7, Biolegend, 1:400), anti-CD62L (MEL-14, Biolegend, 1:400), anti-CD19 (6D5, Biolegend, 1:400), anti-CD45R/B220 (RA3-6B2, Biolegend, 1:400), anti-Fas (SA367H8, Biolegend, 1:400), anti-GL-7 (GL7, Biolegend, 1:400), anti-CD138 (281-2, Biolegend, 1:400), anti-CTLA4 (UC10-4B9, Biolegend, 1:400), anti-GITR (DTA-1, Biolegend, 1:400), anti-ICOS (7E.17G9, Biolegend, 1:100 or 1:400), anti-TIGIT (1G9, Biolegend, 1:400), anti-KLRG1 (2F1/KLRG1, Biolegend, 1:400), anti-ST2 (DIH9, Biolegend, 1:200), anti-IL-17A (TC11-18H10.1, Biolegend, 1:500), anti-IFN-γ (XMG1.2, Biolegend, 1:100), anti-JunB (C-11, Santa Cruz Biotechnology, 1:200), anti-IRF4 (IRF4.3E4, Biolegend,1:100), anti-BATF (D7C5, Cell Signaling Technology, 1:400), anti-Foxp3 (150D, Biolegend, 1:100), anti-GATA3 (16E10A23, Biolegend,1:200), anti-RORgammat (Q31-378, BD, 1:100), anti-T-bet (4B10, Biolegend, 1:100), anti-Helios (22F6, Biolegend, 1:100), anti-CD45.1 (A20, Biolegend, 1:400), anti-CD45.2 (104, Biolegend, 1:400), anti-Ki-67 (16A8, Biolegend, 1:400), anti-goat IgG (Poly4053, Biolegend, 1:100 or 1:400), and anti-rabbit IgG (Poly4064, Biolegend, 1:100 or 1:400).
Validation	Validation data are available on the manufacturer's website and data sheet.

Animals and other organisms

Policy information about studies involving animals; ARRIVE guidelines recommended for reporting animal research

Laboratory animals	Junbfl/fl mice were described previously42. Foxp3Cre (Foxp3YFP-Cre; stock# 016959), Cd4Cre (stock# 017336), Rag1-/- (stock# 002216), and B6SJL (stock# 002014) mice were obtained from the Jackson Laboratory. All mice were maintained on a C57BL/6 background under specific pathogen-free conditions. Sex-matched, 6-12-week-old mice were used for experiments. All animal experimental protocols were approved by the Animal Care and Use Committee at Okinawa Institute of Science and Technology Graduate University.
Wild animals	The study did not involve wild animals.
Field-collected samples	The study did not involve samples collected from the field.

ChIP-seq

Data deposition

☒ Confirm that both raw and final processed data have been deposited in a public database such as GEO.

☒ Confirm that you have deposited or provided access to graph files (e.g. BED files) for the called peaks.

Data access links<br>*May remain private before publication.*	https://www.ncbi.nlm.nih.gov/geo/query/acc.cgi?acc=GSE121295
Files in database submission	GSM3430972 WT_Treg_aJunB.fastq.gz<br>GSM3430973 WT_Treg_aBATF.fastq.gz<br>GSM3430974 WT_Treg_aIRF4.fastq.gz<br>GSM3430975 KO_Treg_aIRF4.fastq.gz<br>GSM3430972_Treg_WT_JunB.bed.gz<br>GSM3430973_Treg_WT_BATF.bed.gz<br>GSM3430974_Treg_WT_IRF4.bed.gz<br>GSM3430975_Treg_KO_IRF4.bed.gz
Genome browser session<br>(e.g. UCSC)	No longer applicable

Methodology

Replicates	All ChIP-seq experiments were performed 2 times.
Sequencing depth	20 million pair-end reads for each experiment
Antibodies	anti-JunB (210, Santa Cruz, 2 µg per ChIP), anti-BATF (WW8, Santa Cruz, 2 µg per ChIP), and anti-IRF4 (M-17, Santa Cruz, 2 µg per ChIP) were used.
Peak calling parameters	Basecalls performed using RTA2<br>ChIP-seq reads were aligned to the mm9 genome assembly using Bowtie2.1.2 with optional parameters "-a --strata --best -m 1 -v 2"<br>peaks were called using Homer v4.10 with the default setting<br>Genome_build: mm9
Data quality	We repeatedly performed ChIP-PCR to comfirm ChIP-seq data quality.
Software	Bowtie2, Homer

Flow Cytometry

Plots

Confirm that:

☒ The axis labels state the marker and fluorochrome used (e.g. CD4-FITC).

☒ The axis scales are clearly visible. Include numbers along axes only for bottom left plot of group (a 'group' is an analysis of identical markers).

☒ All plots are contour plots with outliers or pseudocolor plots.

☒ A numerical value for number of cells or percentage (with statistics) is provided.

Methodology

Sample preparation	Cells were isolated from spleens, lymph nodes, and thymuses by mashing them through cell strainers (BD; 352340). Cells were

Sample preparation	also isolated from lungs and colonic lamina propria using a Lung Dissociation kit (Miltenyi; 130-095-927) and a Lamina Propria Dissociation kit (Miltenyi; 130-397-410), respectively, according to the manufacturer's instructions.
Instrument	BD LSRFortessa, BD FACSAria II, BD FACSAriaIII
Software	BD FACS software was used for collection and FlowJo v10 was used for analysis.
Cell population abundance	2x10^5 -2x10^6 cells per sample were obtained.
Gating strategy	For the all data analysis, FSC-A/SSC-A gating was performed for cell size and characteristics determination and exclusion of debris. In addition, FSC-H/FSC-W gating and SSC-H/SSC-W gating were performed for the exclusion of cell doublets. Then dead cells were excluded by Zombie-NIR staining. Positive gate and negative gate were determined by using isotype control or knockout control. Gating strategies are provided in Supplementary Figure 10.

☒ Tick this box to confirm that a figure exemplifying the gating strategy is provided in the Supplementary Information.
